# Quality of life in Prolactinoma: A systematic review

**DOI:** 10.1007/s11102-024-01392-1

**Published:** 2024-04-24

**Authors:** Mendel Castle-Kirszbaum, Nienke Biermasz, Jeremy Kam, Tony Goldschlager

**Affiliations:** 1 Department of Neurosurgery, Monash Health, 246 Clayton Road, Clayton, VIC 3168 Australia; 2 Department of Surgery, Monash University, Melbourne, Australia; 3 Department of Medicine, Division of Endocrinology, Center for Endocrine Tumors Leiden, Leiden University Medical Center, Leiden, The Netherlands

**Keywords:** Prolactinoma, Lactotroph, Quality of life, Wellbeing, QOL

## Abstract

**Background:**

Prolactinomas are common tumours that significantly reduce quality-of-life (QOL) due to sellar mass effect, secondary hypogonadism, and the peripheral effects of prolactin. Understanding the factors that influence QOL would provide insights into therapeutic targets to optimise patient outcomes and improve wellbeing in prolactinoma.

**Methods:**

A systematic review was performed in accordance with the PRISMA statement. Studies that reported patient QoL using validated metrics were included. Bias and methodological rigour were assessed using the MINORS criteria.

**Results:**

A total of 18 studies were identified studies were available for review, comprising 877 patients. Most were small cross-sectional studies at high risk of bias. Prolactinoma exhibit worse QOL than healthy controls, particularly mental and psychosocial wellbeing. QOL is also worse than patients with non-functional adenomas, but better than those with Cushing’s disease and acromegaly. QOL correlates with prolactin levels, and approaches population baseline with prolonged biochemical control. Dopamine agonists and surgery both improve overall QOL, however improvements are more rapid with surgery.

**Conclusion:**

Poor quality of life in prolactinoma is multifactorial, related to biochemical control, side effects of therapy, and sellar mass effect. Targeting persistent symptoms, reducing healthcare costs, and reducing side-effects of therapy are avenues to improving QOL in patients with prolactinoma.

**Supplementary Information:**

The online version contains supplementary material available at 10.1007/s11102-024-01392-1.

## Introduction

Prolactinoma (functional lactotroph pituitary neuroendocrine tumours) are the most common pituitary adenoma encountered in daily practice. These tumours come to clinical attention due to sellar mass effect, secondary hypogonadism, and the peripheral effects of prolactin. Through these mechanisms the tumour, and its treatment, can cause significant detriment to physical, social, and emotional functioning. Understanding these effects on patient reported quality of life (QOL) would provide insights into therapeutic targets to optimise patient outcomes [1]. Here, we review the literature on QOL in patients with prolactinomas to elucidate the patient and therapeutic factors that influence patient wellbeing.

## Methods

A systematic search of the literature was conducted using the Medline and PubMed databases in accordance with the PRISMA statement [2]. The search included all studies form the database inception until December 2023 using the search string:

*(Prolactinoma (MP) OR Lactotroph OR Hyperprolactinaemia OR Hyperprolactinemia) AND (quality of life OR wellbeing OR QOL)*.

Exclusion criteria were single case reports, studies published in languages other than English, and studies of multiple tumour types where data specific to prolactinomas could not be extracted. The references of identified studies, as well as relevant textbooks, were consulted to identify additional eligible studies.

Titles and abstracts of identified studies were then screened. For appropriate studies, full-text review was performed to determine suitability for inclusion. Inclusion criteria were defined as: (1) Randomized trials, non-randomized trials, and cohort studies that report QOL in prolactinoma; (2) An age-appropriate, validated QoL metric was used to quantify QOL.

Included studies underwent independent data extraction, including study year, study size, treatment status, QOL metric, and QOL outcome. Included studies were assessed for methodological bias using the Methodological Index for Non-Randomized Studies (MINORS) [3].

## Results

A total of 18 studies were identified from the systematic search of the literature (Supplementary Fig. 1), comprising 877 patients [4–20] (Table 1). Risk of bias results are presented in Supplementary Table 1.

**Table 1:** – Studies assessing Quality of life in patients with prolactinoma

Author (Year)	N (Active vs. Controlled disease at start of study)	QoL Metric	Comparison	Design	Outcome
Andela (2016)	92 (0/92)	EQ-5D, SF36, MFI-20, HADS, LBNQ-Pituitary	Other pituitary adenomas	Cross sectional	Prolactinoma had less physical and cognitive complaints, social functioning issues, than CD No difference compared to other adenomas
Athanasoulia (2012)	86 (NR)	EPQ, TPQ	NFPA (*n* = 58), healthy controls (*n* = 172)	Cross sectional	Prolactinoma had increased neuroticism, fear of uncertainty, fatigability and asthenia compared to healthy controls Prolactinoma demonstrated reduced extraversion and increased shyness with strangers compared to NFPA
Baird (2003)	22 (NR)	SIP	Other adenoma types	Cross sectional	Prolactinomas had less impairment than other pituitary tumours, but the characteristics of the impairments were similar
Buckman (1985)	10 (10/0)	BDI, KDS, HRDS	bromocriptine vs. placebo	6 week double bind crossover trial	Depression, anxiety, wellbeing and friendliness scores improved with bromocriptine and approached values seen in healthy controls Global scores of wellbeing and libido improved with bromocriptine.
Castle-Kirszbaum (2022)	18 (18/0)	ABSQ, SNOT-22	Other pituitary adenomas	Prospective cohort with 12 month follow-up	QOL increased from baseline levels by 3mo postop and continued to improve at 6mo and 12mo Prolactinoma increased in QOL more than other adenoma types
Cesar de Oliveira Naliato (2008)	50 (25/25)	SF-36	Healthy controls	Cross sectional	QoL scores worse in all SF-36 components compared to controls No difference in scores between cabergoline and bromocriptine Patients with normalised prolactin had better scores than patients with persistent hyperprolactinaemia in all components General health, social functioning, and mental health were all worse in patients with normalised prolactin compared to controls Amenorrhoea was not associated with SF-36 scores SF-36 Physical role, pain and mental health scores correlated with PRL levels
Ernernsson (2023)	32 (NR)	FIS, ESS, SF-36	Normative data	Cross sectional	QoL scores worse in all SF-36 components compared to controls. 37.5% had day-time sleepiness (ESS > 10)
Heald (2004)	24 (NR)	HADS, WHO-QoL, GHQ, FACT, SAS	Other adenoma types	Cross sectional	
Johnson (2003)	39 (NR)	SF-36	Other adenoma types Healthy controls	Cross sectional	Mental health scores, and vitality lower in prolactinoma compared to controls More impairment in mental than physical aspects of QOL
Kars (2007)	55 (18/37)	SF-36, NHP, MFI-20, HADS	Healthy controls	Cross sectional	Anxiety and depression (HADS) and fatigue (MFI-20) worse with prolactinoma Social functioning and role limitations due to physical problems of the SF-36 Energy, emotional reaction, and social isolation subscales of the NHP worse in prolactinoma
Leistner (2015)	74 (24/50)	PSQI, Euro-QoL, BDI	Healthy controls Other pituitary adenomas	Cross sectional	Cushing’s patients had worse QOL and depression scores than prolactinomas
Lobatto (2019)	16 (16/0)	Euro-QoL, SF-36, LBNQ-Pituitary, VFQ-25, SNOT-22, ASK-12, HLQ	Other pituitary adenomas	Prospective cohort with 6 month follow-up	After surgery: 81% improved in LBNQ-Pituitary score as early as 5d post-op Clinically significant improvement in SF-36 mental in 56%, and 37.5% in physical. Mental SF-36 improved within 6w 50% clinically significant improvement in EQ-index after 6mo Sinonasal morbidity was at baseline by 6w after surgery
Raappana (2012)	17 (6/11)	15D	Healthy controls	Cross sectional	Prolactinoma with DA treatment had a lower sexual activity scoresw Worse scores in sleep mental health, sex life, vitality, and distress in prolactinoma compared to control
Reavley (1997)	65 (65/0)	HADS, SCL90	NFPA and acromegaly	Cross sectional	54% had definite or borderline anxiety Similar rates of depression, higher rates of hostility in prolactinoma
Ritvonen (2014)	26 (4/22)	15D	Other adenoma types Healthy controls	Cross sectional	Scores similar to controls
Van der Klaauw (2008)	128 (NR)	SF-36, NHP, MFI-20, HADS	Other adenoma types Healthy controls	Cross sectional	PRL scores worse in all scales cf. controls (mean 0.7 SD above control) QoL parameters did not differ between microadenomas and macroadenomas and those on DA and not
van der Meulen (2021)	116 (38/78)	LBNQ-Pitutiary, SF-36, Euro-QoL	nil	Cross sectional	Association between higher healthcare utilisation, worse QOL scores and higher prolactin levels
Vega-Beyhart 2019	53 (28/25)	SF-36	Healthy controls	Cross sectional	Persistent hyperprolactinaemia had worse SF-36 scores than those with biochemically controlled disease Prolactin levels varied inversely with Mental and physical SF-36 cores Mental scores were lower in patients with hyperprolactinemia, VF deficits, and macroadenomas Physical scores were lower in patients with hyperprolactinemia, VF deficits, and central adrenal insufficiency

ABSQ = Anterior skull base questionnaire; ASK-12 = Anterior Skull Base Nasal Inventory; BDI = Beck Depression Inventory; EPQ = Eysenck personality questionnaire; ESS = Epworth Sleepiness Scale; Euro-QoL = European QoL; FACT = Functional Assessment of Cancer Therapy; FIS = Fatigue Impact Scale; GHQ = General Health Questionnaire 28; HADS = Hospital Anxiety and Depression Scale; HLQ = Health and Labor Questionnaire; HRSD = Hamilton depression score; KDS = Kellner distress scale; LBNQ-Pituitary Leiden Bother and Needs Questionnaire Pituitary; MFI-20 = Multidimensional Fatigue Inventory; NHP = Nottingham Health Profile; PSQI = Pittsburgh Sleep Quality Index; SAS = Modified Social Adjustment Scale; SCL-90 = 90-item symptom checklist; SF-36 = Short Form 36; SIP = Sickness Impact Profile; SNOT-22 = Sinonasal Outcome Test; TPQ = Tridimensional Personality Questionnaire; VFQ-25 = Visual Function Questionnaire; WHO-QoL = World Health Organization Quality of Life Scale

The literature consisted of small retrospective and prospective cohort studies, spanning from 1985 to 2023. These were generally at a high risk of bias. Most studies (81%, 13/16) were cross-sectional, while the three prospective interventional studies had an overall short follow up, ranging from 6 weeks to 12 months. Of those studies reporting disease activity, only 42.6% (252/592) of patients had hyperprolactinemia at the time of the study.

QoL was measured with several metrics including sinonasal and skull base metrics (Anterior skull base questionnaire (ABSQ) [21], Anterior Skull Base Nasal Inventory (ASK-12) [22], Leiden Bother and Needs Questionnaire Pituitary (LBNQ-Pituitary) [4], Sinonasal Outcome Test (SNOT-22) [23], Visual Function Questionnaire (VFQ-25) [24]); depression and anxiety metrics (Beck Depression Inventory (BDI) [25], Hospital Anxiety and Depression Scale (HADS) [26], Hamilton depression score (HRSD) [27], Kellner distress scale (KDS) [28]); fatigue and sleep quality metrics (Epworth Sleepiness Scale (ESS) [29], Fatigue Impact Scale (FIS) [30], Multidimensional Fatigue Inventory (MFI-20) [31], Pittsburgh Sleep Quality Index (PSQI) [32]); social functioning and personality metrics (Eysenck personality questionnaire (EPQ) [33], Modified Social Adjustment Scale (SAS) [34], Tridimensional Personality Questionnaire (TPQ) [35]); and Global QOL metrics (Pittsburgh Sleep Quality Index (PSQI) [32], Functional Assessment of Cancer Therapy (FACT) [36], General Health Questionnaire (GHQ) [37], Health and Labor Questionnaire (HLQ), Nottingham Health Profile (NHP) [38], Short Form 36 (SF-36) [39], Sickness Impact Profile (SIP) [40], World Health Organization Quality of Life Scale (WHO-QoL) [41], 90-item symptom checklist (SCL-90) [42], and 15D [43]).

### Prolactinoma compared to Healthy Controls

Ten studies compared patients with prolactinoma to healthy controls. Patients with prolactinoma had worse overall QOL [9–11, 18] and mental health [9]. They also experienced greater fatiguability [5], distress [15], and fear of uncertainty [5]. Overall, patients with prolactinoma tended to demonstrate greater impairment in mental rather than physical components of QOL [11, 12]. Anxiety, depression, and mood disorders were more prevalent in patients with prolactinoma, with more than half exhibiting definite or borderline anxiety [16]. Sleep quality and duration was also poorer in patients with prolactinoma [15], with more than one-third demonstrating excessive daytime sleepiness [10].

### Compared to other pituitary adenomas

Eleven studies compared QOL in patients with prolactinoma to patients with other pituitary adenomas. Prolactinomas exhibited better overall QOL than patients with Cushing’s disease [8, 44], including physical and cognitive complaints [4], psychosocial functioning [4, 44], and depression scores [13].

Compared to patients with non-functioning adenomas (NFPA), patients with prolactinoma demonstrated a more reserved personality type, characterised by reduced extraversion and increased shyness with strangers [5]. Depression rates were similar, but those on dopamine agonist (DA) therapy showed greater hostility scores [16].

In one study, QOL scores were 0.7 standard deviations (SD) below healthy controls, slightly worse than NFPA (0.5 SD) but better than Cushing’s (1.1 SD) and acromegaly (1.4 SD) [18]. Although this hierarchy of QOL impairment is consistent in treatment naïve patients [11], in surgical series where prolactinoma are usually larger and resistant to medical therapy, QOL detriment can approach [8] or exceed [14] Cushing’s disease and acromegaly patients.

### Effect of treatment on QOL

Mental and physical QOL scores correlated inversely with prolactin levels [9, 20]. In many cases biochemical control was not associated with a return of QOL to that of healthy controls, except when biochemical control had been achieved for several years [17, 20]. SF-36 scores were 20–25% lower in patients with active disease compared to those with biochemical control [20]. There is no clear difference in QOL between medical or surgical treatment modalities [19].

In one small double-blind crossover study, treatment with DA led to improvements in depression, anxiety, wellbeing, and friendliness scores as prolactin levels declined [7]. DA therapy improved libido by reducing prolactin [7], but the therapy itself may impact sexual health, as in patients with similar prolactin levels, the use of DA was associated with worse sexual activity scores [15]. In one small study there was no clear difference in QOL between patients treated with bromocriptine and cabergoline [9].

Surgery was associated with improved QoL as early as 5 days postoperatively [14], and scores continued to improve throughout the first postoperative year [8]. Prolactinoma, more than any other adenoma type, had the greatest improvement of QOL within one year of surgery [8, 14]. Clinically significant improvements in SF-36 mental and physical scores were seen in 56% and 37% respectively within 6 months of surgery, although improvements were often seen earlier [8, 14]. Only 6% of cases had clinically significant worsening of QOL after surgery [14].

### Predictors of QOL in Prolactinoma

In addition to higher prolactin levels, visual field deficits and central adrenal insufficiency also predicted worse QoL [20]. Amenorrhoea was not associated with QOL, while the association of QOL and adenoma size was conflicting [18, 20]. Patients with higher prolactin and worse QOL scores also had greater healthcare utilisation and spent more money on healthcare [19].

## Discussion

Patients with prolactinoma exhibit worse QOL than healthy controls, particularly mental and psychosocial wellbeing. Compared with other functional adenomas, QOL is less severely affected, while QOL is worse than those with NFPA. QOL correlates with prolactin levels, and approaches population baseline with prolonged biochemical control. The effects of DA are beneficial due to suppressive effects on prolactin, however they may themselves contribute to psychosocial dysfunction. Surgery provides a rapid, substantial improvement to QOL, likely though inducing biochemical control and freedom from DA as well as immediate relief of sella mass effect. Relief of mass effect, biochemical control of hyperprolactinaemia, and freedom from DA appear key to normalising QOL in patients with prolactinoma.

### Putative effects of Hyperprolactinemia on QOL

Classically confined to the initiation and maintenance of lactation in females, it has become clear that prolactin exerts a range of metabolic, immunologic, and reproductive effects.

Hypogonadotrophic hypogonadism is the most common presenting symptom of hyperprolactinemia, manifesting as oligo-amenorrhoea in pre-menopausal women and impotence in males. Hyperprolactinemia reduces the frequency and amplitude of GnRH secretory pulses, and downstream reductions in LH secretory pulses lead to gonadal suppression. Prolactin exerts its inhibitory tone on kisspeptin neurons, which are the key regulators of pulsatile GnRH neuronal secretion [45]. This is evidenced by the restoration of gonadal function with kisspeptin supplementation in patients with hyperprolactinemia induced hypogonadism [46, 47]. In women, hypogonadism is associated with sexual dysfunction, fatigue, sleep and mood disturbances with corresponding reductions in QOL [48]. In men, the negative effects of hypogonadism on vitality [49], body composition [50], mood [51], cognitive function [52, 53] and sexual health lead to similarly reduced QOL.

Hyperprolactinemia may affect QOL independent of its effects on gonadal hormones. Hyperprolactinaemia is associated with anxiety, somatization, hostility and depression [54]. This may be augmented by the emotional impact of their diagnosis and variable adoption of the sick role [55, 56]. The mechanisms of direct effects of prolactin on the brain and behaviour are unclear. Prolactin influences hypothalamic appetite regulation by inducing leptin resistance, inducing hyperphagia and weight gain [57]. Hypogonadism, particularly androgen deficiency, may further contribute to the development of the metabolic syndrome in hyperprolactinemia. It also acts peripherally, inducing insulin resistance [58, 59] and dyslipidaemia [60]. Dissatisfaction with body composition and appearance may affect mood and QOL. Hyperprolactinemia also has direct effects on bone metabolism, increasing resorption and inhibiting new bone formation, predisposing to fractures [61, 62], which may affect physical functioning, pain, and QOL [63].

Headache has a significant impact on QOL in patients with pituitary adenoma, and prolactinoma may be particularly cephalalgogenic [64, 65]. Headaches occur in both micro- and macro-prolactinomas [66], may be induced by TRH (a potent prolactin secretagogue) [67], and improve with DA therapy [68], implicating prolactin in headache pathogenesis.

### Dopamine agonists and QOL

DA are the primary treatment modality for prolactinoma due to their efficacy in achieving biochemical control with a relatively well tolerated side effect profile. Improvements in hyperprolactinemia [7], sexual function [69], metabolic syndrome [70], insulin resistance [71], and restoration of normal gonadal function [72] likely underlie the improvements in QOL seen with DA therapy. However, even when biochemically normalised, QOL does not reach that of healthy controls, suggesting DA themselves may impact QOL, although persistent mass effect or a detrimental effect of hypoprolactinemia^64^ remain considerations. Treatment duration did not appear to influence QOL [9].

Common side effects of DA include nausea, dizziness and vertigo, headaches, postural hypotension, and abdominal pain [73]. Psychiatric side effects, including mood disorders (depression and mania) and impulse control disorders can also be induced by DA, presumably though stimulation of mesocortico-limbic dopaminergic pathways. These may manifest as gambling, compulsive spending, binge eating, hyperaggressive behaviour, depression, and hypersexuality (augmented by the return of gonadal function), and may occur to a variable degree in a significant proportion of patients [74–76]. These psychiatric effects impair psychosocial function and may be deleterious to relationships, reputation, and QOL.

The cumulative cost of DA and their ongoing monitoring can be significant financial stress for patients. The cost of Cabergoline can be $5 to $50 USD per tablet [77], which can total many thousands of dollars annually if higher than standard dosing is required. Hospitalization and specialist care (endocrinologists, general practitioners, ophthalmologists, neurosurgeons, and mental health clinicians) further contribute to healthcare utilisation and costs, which are associated with worse QOL [19].

### Surgery for prolactinoma and its effect on QOL

Surgery is traditionally indicated for patients with prolactinoma refractory or intolerant to medical therapy, although first-line surgery is highly effective for patients without cavernous sinus invasion [78, 79]. Surgical cohorts typically exhibit worse QOL due to a prolonged disease course, persistent hyperprolactinemia, side effects of high dose DA, more aggressive histology, and prominent sellar mass effect. The sequelae of sellar mass effect on quality of life [8, 80, 81] is well established, with visual dysfunction [82], hypopituitarism [83], and headache [84] correlated to QOL. Surgery rapidly controls hyperprolactinemia, reduces intrasellar pressure and parasellar mass effect, improves headache [85], and often facilitates emancipation from DA therapy [86], leading to substantial and early improvements in QOL. This supports current guidelines recommending surgery as a viable first-line therapy in well-circumscribed prolactinomas without cavernous sinus extension [87].

### Limitations

The included studies were mostly small, cross-sectional, and subject to bias. Of the multiple different QOL metrics were used across studies, few assess the full gamut of symptoms that may occur in prolactinoma and its treatment. The majority of patients in these cross-sectional studies were on DA therapy and had controlled disease. Data was rarely stratified by disease activity, treatment modality, adenoma volume, or socioeconomic status, limiting comparisons. No study directly compared medical and surgical QOL outcomes.

## Conclusions

Prolactinomas are associated with decreased QOL due to sellar mass effect, hyperprolactinemia, ensuing hypogonadism, and the adverse effects of DA therapy. Detriments to QOL are global, but mental health, sexual function, and psychosocial functioning appear particularly affected. QOL in patients with prolactinoma is significantly worse than healthy controls, slightly worse than those with NFPA, but often better than those with Cushing’s disease or Acromegaly. Resolution of hyperprolactinemia improves QOL, but slight residual impairment generally persists due to persistent mass effect or the adverse effects and healthcare burden of medical therapy. With long term biochemical control, QOL can approach that of healthy controls. Targeting persistent symptoms, reducing healthcare costs, and reducing side-effects of therapy are avenues to improving QOL in patients with prolactinoma.

### Electronic supplementary material

Below is the link to the electronic supplementary material.

Supplementary Material 1

Supplementary Material 2

## Data Availability

No datasets were generated or analysed during the current study.

## Code availability

N/A
