# Supplementary material for: Quality of life in Prolactinoma: A systematic review
Source: Pituitary. 2024 Apr 24;27(3):239–47. doi: 10.1007/s11102-024-01392-1 (PMC11150290; doi:10.1007/s11102-024-01392-1)
Supplement: Supplementary file 1 — Supplementary Material 1 [file 11102_2024_1392_MOESM1_ESM.docx]

**Supplementary Table 1 - MINORS criteria for methodological rigour in non-randomised cohort studies**

| Author | Aim | Consecutive | Prospective | Endpoints | Unbiased | Follow-up | Attrition | Study size | Control | Contemporaneous | Baseline Equivalence | Analysis | Total |
| --- | --- | --- | --- | --- | --- | --- | --- | --- | --- | --- | --- | --- | --- |
| Andela (2016) | 2 | 1 | 1 | 2 | 2 | 0 | 0 | 0 | - | - | - | - | 8/16 |
| Athanasoulia (2012) | 2 | 0 | 0 | 2 | 2 | 0 | 0 | 0 | 1 | 1 | 1 | 1 | 10/24 |
| Baird (2003) | 2 | 1 | 1 | 0 | 1 | 0 | 1 | 0 | - | - | - | - | 6/16 |
| Buckman (1985) | 2 | 2 | 2 | 2 | 2 | 2 | 0 | 0 | 2 | 2 | 2 | 2 | 20/24 |
| Castle-Kirszbaum (2022) | 2 | 2 | 2 | 2 | 2 | 1 | 1 | 0 | - | - | - | - | 12/16 |
| Cesar de Oliveira Naliato (2008) | 2 | 0 | 0 | 2 | 2 | 0 | 0 | 0 | 1 | 1 | 1 | 1 | 10/24 |
| Ernernsson (2023) | 2 | 2 | 1 | 2 | 2 | 0 | 0 | 0 | - | - | - | - | 9/16 |
| Heald (2004) | 2 | 0 | 1 | 2 | 2 | 0 | 0 | 0 | - | - | - | - | 7/16 |
| Johnson (2003) | 2 | 0 | 2 | 2 | 2 | 0 | 0 | 0 | - | - | - | - | 8/16 |
| Kars (2007) | 2 | 0 | 1 | 2 | 2 | 0 | 0 | 0 | 1 | 0 | 0 | 0 | 8/24 |
| Leistner (2015) | 2 | 0 | 0 | 1 | 2 | 0 | 0 | 0 | 2 | 1 | 1 | 1 | 10/24 |
| Lobatto (2019) | 2 | 2 | 2 | 2 | 2 | 2 | 2 | 0 | - | - | - | - | 14/16 |
| Raappana (2012) | 2 | 1 | 1 | 1 | 2 | 0 | 0 | 0 | - | - | - | - | 7/16 |
| Reavley (1997) | 2 | 1 | 1 | 1 | 2 | 1 | 1 | 1 | 2 | 2 | 0 | 1 | 15/24 |
| Ritvonen (2014) | 2 | 0 | 1 | 1 | 2 | 0 | 0 | 0 | 2 | 1 | 0 | 1 | 10/24 |
| van der Klaauw (2008) | 2 | 2 | 2 | 2 | 2 | 0 | 0 | 0 | 2 | 1 | 0 | 1 | 14/24 |
| van der Meulen (2021) | 2 | 1 | 1 | 2 | 2 | 0 | 0 | 0 | - | - | - | - | 8/16 |
| Vega-Beyhart 2019 | 2 | 0 | 1 | 1 | 2 | 0 | 0 | 0 | - | - | - | - | 6/16 |

The MINORS criteria are scored as 0 (not reported), 1 (reported but inadequate) or 2 (reported and adequate). For non-comparative studies, a maximal score of 16 can be achieved, based on the following 8 criteria:

- Aim – A clearly stated aim
- Consecutive patients – All consecutive patients that meet inclusion criteria are included
- Prospective – Data is collected prospectively from a pre-defined protocol
- Endpoints – Unambiguous endpoints that correlate with study aim, assessed on an intention-to-treat basis
- Unbiased – Unbiased outcome assessment (e.g. blinded)
- Follow-up - Follow-up period appropriate to detect primary outcome and adverse events
- Attrition - Loss to follow up <5%
- Study size - Prospective calculation of the study size

For comparative studies, an additional 4 criteria are assessed, bringing the maximal achievable score to 24:

- Control – Adequate control group
- Contemporaneous – Control and intervention groups studied at same time (e.g. not historical control)
- Baseline equivalence – Baseline factors are similar between control and intervention groups
- Analysis - Adequate statistical analyses
