# Supplementary figures and images for: Quality of life in Prolactinoma: A systematic review
Source: Pituitary. 2024 Apr 24;27(3):239–47. doi: 10.1007/s11102-024-01392-1 (PMC11150290; doi:10.1007/s11102-024-01392-1)

**Supplemental Fig. 1 – PRISMA flow diagram of included studies**


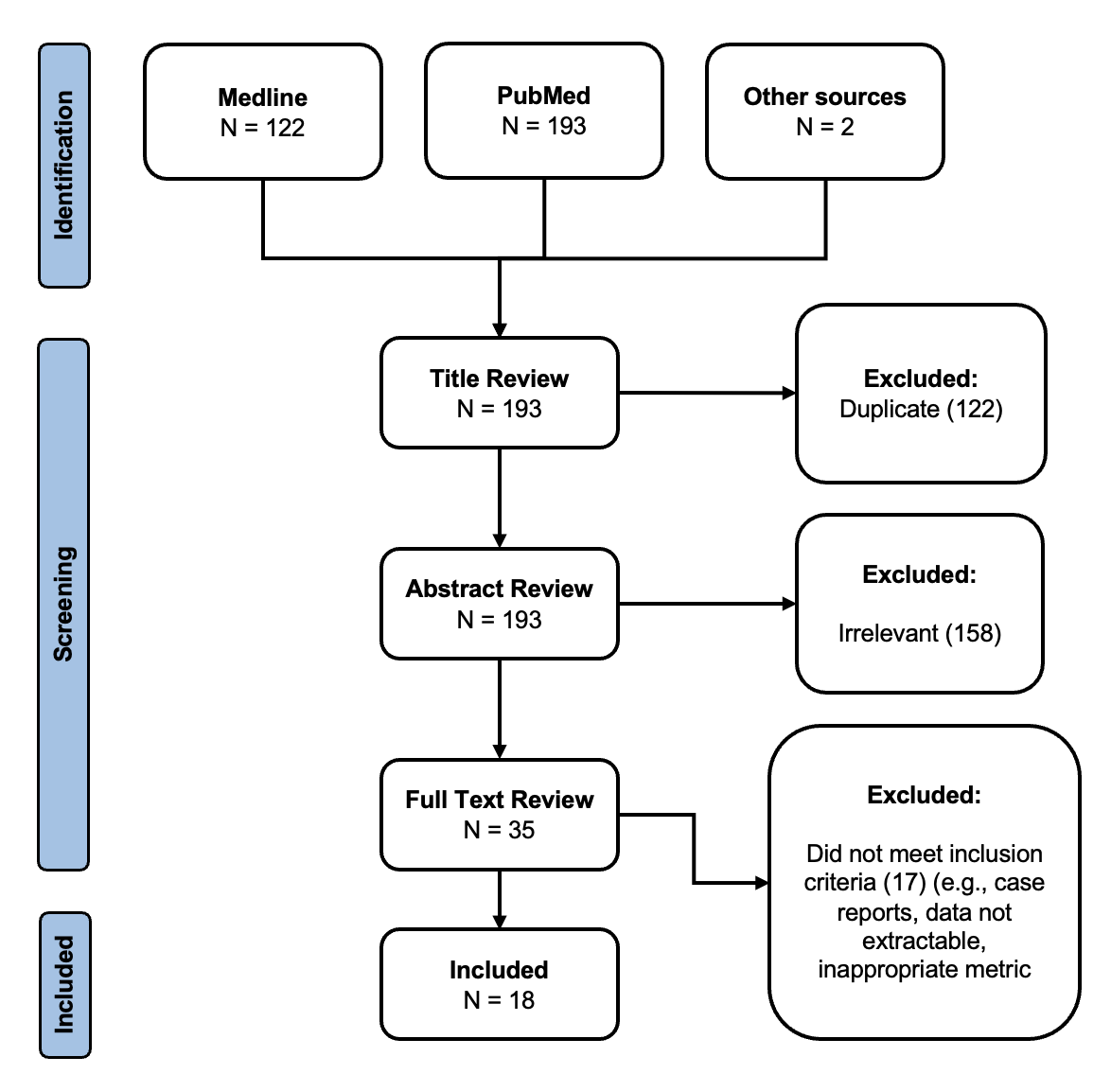

Supplement: Supplementary file 2 — Supplementary Material 2 [file 11102_2024_1392_MOESM2_ESM.docx]
